# Key stakeholder perspectives on the development and real-world implementation of a home-based physical activity program for mothers at risk of postnatal depression: a qualitative study

**DOI:** 10.1186/s12889-021-10394-8

**Published:** 2021-02-16

**Authors:** Megan Teychenne, Maria Apostolopoulos, Kylie Ball, Ellinor K. Olander, Rachelle S. Opie, Simon Rosenbaum, Rachel Laws

**Affiliations:** 1 Deakin University, Geelong, Australia; 2 Institute for Physical Activity and Nutrition (IPAN), School of Exercise and Nutrition Sciences, Deakin University, Melbourne Burwood Campus, 221 Burwood Hwy, Burwood, VIC 3125 Australia; 3 Centre for Maternal and Child Health Research, School of Health Sciences, City, University of London, London, UK; 4 School of Psychiatry, University of New South Wales, Sydney, Australia; 5 Black Dog Institute, Sydney, Australia

**Keywords:** Physical activity, Postnatal depression, Program, Co-design, Implementation, Interviews, CFIR, Research translation

## Abstract

**Background:**

Physical activity (PA) is a modifiable risk factor for postnatal depression (PND) and programs are needed to enhance PA amongst women at risk of PND. Key stakeholder involvement in informing development and implementation of such programs is vital. However, little research demonstrates key stakeholder insights to inform the design and delivery of PA programs for improving PND. The aim of this study was to explore key stakeholder perspectives on the design and delivery of a home-based PA program for mothers with PND symptoms to inform future real-world implementation and scale-up.

**Methods:**

A descriptive qualitative study was undertaken whereby semi-structured interviews were conducted with representatives from various key stakeholder organisations involved in postnatal PA and/or mental health, public health and policy in Australia (*n* = 11). Interviews were conducted between September to November 2019 and explored stakeholder perceptions on the design and delivery of a home-based PA program for mothers with PND symptoms. The interview schedule was informed by both the Consolidated Framework for Implementation Research (CFIR) and the PRACTical planning for Implementation and Scale-up (PRACTIS) guide. Data were analysed thematically using both deductive and inductive coding.

**Results:**

The *relative priority* of PND and PA was high for most organisations involved, although none implemented PA programs supporting women at risk of PND. Most stakeholders perceived the program as *appealing* due to addressing barriers to postnatal PA, although identified some *feasibility* issues regarding funding and delivery mechanisms. Suggestions for program *adaptations* included an equity focus (e.g. providing socioeconomically disadvantaged women with a greater program dose; translating web-app based content into various languages). Planned components of the program were suggested to align (i.e. *relative advantage*) with existing initiatives (e.g. equipment hire for nurseries scheme) and screening systems for PND (timing of referral). Perceived *barriers* to scale-up included logistics/cost of equipment, organisational capacity demands and safety risks/liability. Perceived *enablers* to scale-up included linking the program with ‘adjunct’ programs and services.

**Conclusions:**

While the program was appealing and most organisations could see a role in endorsing and/or referring to the program, funding and delivery mechanisms still need to be identified.

**Supplementary Information:**

The online version contains supplementary material available at 10.1186/s12889-021-10394-8.

## Background

Postnatal depression (the onset of depression up to 12-months after giving birth [1]) affects 13% of new mothers worldwide [2] and can have a severe impact mentally, emotionally, and behaviourally on mother, baby and their families. Although physical activity has been shown to be effective in preventing and treating postnatal depressive symptoms [3, 4], most postnatal women are not meeting recommended levels of physical activity [5]. This is in part due to barriers preventing new mothers from leaving the house (e.g. sleeping and feeding routines) or being able to exercise at any time of day. Thus, home-based physical activity programs have been proposed to help overcome these barriers, and have recently been successfully piloted amongst women experiencing PND symptoms [6]. Recent calls to action by leading researchers in the field and professional organisations (e.g. Exercise Sport Science Australia) have highlighted the need for the implementation of feasible physical activity programs for mental health into routine practice [7], and internationally, organisations are also calling for implementation of perinatal mental health services [8]. Yet, it is unclear as to whether it is feasible for home-based physical activity programs targeting women with PND symptoms to be scaled-up and implemented into real-world settings.

Despite evidence of efficacy, implementation of population physical activity programs is challenging, given the variability of factors influencing success (e.g. resources, infrastructure, values of participants and stakeholders) [9, 10]. Although a previous pilot study suggested high acceptance and uptake by women with PND symptoms [6], the current study builds on this evidence-base by exploring the views of organisations that are likely to be involved in the delivery of such an intervention in practice. To increase the likelihood of successful implementation and scale-up of population physical activity programs, it is important that key organisational stakeholders potentially involved in the implementation of the program have input into intervention design and delivery [10]. Engaging stakeholder organisations can provide essential insights regarding the capacity and potential role of organisations in supporting program implementation (e.g. funding, endorsing, referring) barriers and facilitators of delivering the program in routine service delivery and strategies and actions (including suggested changes/alterations) of proposed programs to ensure the intervention is scalable and sustainable in real world practice. Yet, this important step is not often completed by researchers and thus there is currently a lack of evidence that provides stakeholder perspectives to inform scale-up and implementation of home-based physical activity programs, particularly for reducing the risk of PND.

The PRACTical planning for Implementation and Scale-up (PRACTIS) guide can be used to guide the process of translating evidence-based physical activity interventions into practice [10]. The guide outlines four steps for planning dissemination and implementation: *Step 1)* Characterise parameters of the implementation setting (e.g. intervention population, implementers, delivery setting/organisation); *Step 2)* Identify and engage key stakeholders across multiple levels within delivery systems (e.g. intervention funding/responsibility, dissemination, host, user); *Step 3)* Identify contextual barriers (i.e. the physical, social, cultural environment whereby the intervention will be integrated) and facilitators to implementation (e.g. individual-, provider-, organisational- and community/systems-level); *Step 4)* Address potential barriers to effective implementation (through formative and process evaluation) [10]. Stakeholders can and should be involved in all four steps. Yet, there is little published literature on key stakeholder perspectives to inform the design and delivery of physical activity programs (particularly home-based programs) for preventing and/or managing PND symptoms [11].

The aim of this study was to explore key organisational stakeholder perspectives on the design and delivery of a home-based physical activity program for postnatal women with depressive symptoms (*Mums on the Move*) to inform future real-world implementation and scale up.

## Methods

### Study design

This study was informed by pragmatic research paradigm that typically applies research methods most suitable to answering the research problem [12]. In this case, qualitative descriptive methods [13] using individual interviews with thematic analysis [14, 15] was considered an appropriate approach to gain an initial understanding of stakeholder views on the design and delivery of the *Mums on the Move* program. This formative research was part of a stakeholder engagement strategy of involving end users in planning for intervention scale up, shown to be important in promoting program uptake in real world settings [10]. A pragmatic approach was well suited to this study of understanding the practical issues or potential problems of delivering this program at scale within existing service and policy contexts. In line with a pragmatic approach [16], the authors acknowledge that the results presented have been social constructed between researchers and participants and that individuals beliefs, experiences and context are central in shaping the perspective presented. As such the researchers consider themselves part of the knowledge generation process and not separate from it which has been further acknowledged under ‘researcher reflexivity’. To enhance methodological rigour and transparency, reporting follows the Standards for Reporting Qualitative Research (SRQR) [17].

### Recruitment

In September to November 2019, representatives from selected key stakeholder organisations were recruited into this study. Key stakeholder organisations were selected based on having potential involvement in implementation of postnatal physical activity and/or mental health programs such as healthcare professionals, mental health professionals, community organisations supporting new mothers, and government and organisations involved in mental and/or perinatal health policy and practice. The researchers aimed for maximum variation sampling to recruit a range of health care professionals and advisors, representing various job roles (e.g. policy makers, practitioners, service delivery managers) and organisational perspectives (government agency, professional association, NGO, Hospital/clinical/community health service) relevant to implementation of the intervention into real world practice. No additional sampling criteria beyond role was used during this stage. We acknowledge that other sampling criteria (e.g. years of experience in this field) could have been considered during recruitment.

Most stakeholder organisations approached (10/16) were local (Melbourne, Australia) or state-based, whilst the remainder (*n* = 6/16) were national organisations. Firstly, stakeholder organisations and representatives were identified through members of the author’s research institution who had established links, including the research institutes Stakeholder Relationships Manager (12 out of 16). For relevant organisations without existing professional relationships, an appropriate person/representative from the organisation was identified via a web-search (4 out of 16 identified this way). Secondly, identified stakeholder representatives were emailed a description of the research project and consent form (*n* = 16). In some cases, this information was referred on by those representatives to colleagues within the selected organisation (based on the relevance of their position and time capacity). Upon indicating interest in participating in the study, an interview time was organised with the research team.

### Data collection

Data collection involved either face-to-face or semi-structured telephone interviews, conducted one-on-one or as a small group (based on participant preference). Interviews explored stakeholder perceptions on the design and delivery of a home-based physical activity program for mothers with PND symptoms (*Mums on the Move*). The lead author (MT) conducted the interviews between September and November 2019 and were audio-recorded with permission. A semi-structured interview schedule was developed, informed by both the Consolidated Framework for Implementation (CFIR) [18] and the PRACTIS guide for implementation and scale up of physical activity interventions [10] (see Supplementary Table 1). The CFIR is a widely used implementation framework that provides a range of constructs associated with effective implementation based on 500 published sources across 13 scientific disciplines [18]. The PRACTIS guide provides a practical guide for researchers on evidence-based strategies to improve research-practice translation in the scale up of physical activity interventions in practice contexts [10]. The CFIR and PRACTIS guide were used together to ensure issues likely to be important to implementation and translation as identified in the literature were included in the interview schedule. The resulting interview schedule explored aspects including, but not limited to: 1) How the program *fits* with existing policies, initiatives, programs and delivery systems; 2) What *role* the organisation (or other suggested organisations) may play in either *funding, endorsing, implementing* the program in a potential real-world roll out; 3) *Feasibility* of, and *barriers and facilitators* to, the program being implemented at scale; 4) *Changes/alterations* suggested to make the program fit/work more effectively in real-world settings; 5) *Relative advantage* – how the program compares to other existing programs. The interviews were also informed by background research on each organisation (including current policies, initiatives, priorities) to ensure the interview schedule was well-informed and prompts could be tailored to each stakeholder organisation, but with enough commonalities to generate broad insights across key questions. The interview schedule was pilot tested and refined with the research institutes Stakeholder Relationships Manager.

### The *Mums on the Move* program

The program on which interviews were based around is *Mums on the Move* – a multi-component, home-based physical activity program, which has been pilot-tested and found acceptable and well-liked by women [6]. Briefly, the program aims to increase physical activity and reduce PND risk among postpartum women (3–9 months postpartum) with heightened depressive symptoms. The intervention, underpinned by the social ecological model [19] proposes: 1) provision of free home exercise equipment (treadmill or exercise bike) for 3-months (physical environmental domain), 2) 6-month access to purposely designed web-app for knowledge building, assisting with overcoming barriers to physical activity and for self-monitoring and goal setting (intrapersonal domain), 3) 6-month access to online social support forum and SMS prompts* (*an additional component not included in initial pilot study) for enhancing social support (social domain). A brief summary of the program was provided to stakeholders prior to their interview.

### Data analysis

Interviews were audio-recorded and transcribed verbatim. The interviewer (MT) took detailed notes throughout the interviews and these notes were reviewed alongside the interview transcripts. Thematic analysis following phases outlined by Braun & Clarke [14, 15] was performed by MA. The first step consisted of familiarisation with the data through repeated reading of interview transcripts and notes (Phase 1). Coding was initially undertaken deductively guided by the ideas and concepts within the Consolidated Framework for Implementation Research (CFIR) [18]. When the data did not fit with this coding framework, codes were created from the data (inductive coding) (e.g. barriers associated with the program) (Phase 2). The next step involved searching for themes and combining similar sub-categories (e.g. logistics and cost of equipment) to create major categories (e.g. equipment barrier) (Phase 3). Following this, themes were reviewed (e.g. equipment) (Phase 4), clearly defined and named (e.g. barriers to scale up) (Phase 5). The final step involved write-up of results (Phase 6). Written participant quotes were selected to provide a concise summary of key themes with these anonymised using an assigned number (i.e. Stakeholder, 1) and broadly described as ‘policy advisor’ or ‘practitioner/service manager’ in order to protect participant identity. NVivo version 12.0 qualitative data analysis program was used to code and organise the data. At the beginning of Phase 2, in order to explore various possible interpretations of the data a random sub-set of two transcripts were independently coded by a second author (MT). Both coders then met and engaged in in-depth discussions of their interpretations of the data. Although some terminology differed slightly, no major discrepancies in interpretation were identified. Both MT and MA were involved in in reviewing and naming the final themes.

### Researcher reflexivity

MT (PhD) is a behavioural epidemiologist conducting research in the field of physical activity and mental health, with an interest in developing behavioural interventions to improve mental health and wellbeing of populations. MT developed the Mums on the Move program and has led initial pilot testing of this program and is also a mother of young children. She secured funding for this stakeholder perspectives project and undertook the interviews with participants. We acknowledge there is the potential for social desirability bias given the interviewer was also the designer/ developer of the intervention. Steps taken to reduce social response bias included: 1) At the beginning of each interview, it was emphasised to participants that researchers were only interested in their honest thoughts and perspectives on the scale up of the program within existing service delivery context and that there were no right or wrong answers; 2) Participants were assured no identifying information (people or organisations) would be published/presented. Stakeholders (participants) were not known to MT, except for one. MT’s intimate knowledge of the program enabled her to probe or ask questions during interviews to acquire greater detail from participants (compared to an interviewer who was not involved so intimately in the design and development of the program). Further, as a mother to young children, MT understands the barriers to postpartum physical activity well, which may have played into the process into the types of questions she probed and responded to during interviews.

MA (BExSportSci (Hons)) was the project manager and also undertook data analysis. She has previous experience in qualitative data analysis and has conducted research in the field of physical activity and postnatal women. Both MT and MA have not worked in clinical/ community practice nor have been involved in program scale-up implementation from a practice/ service perspective. Remaining co-authors were not directly involved with the interviews and analysis however, they were utilised as a sounding board whereby they had the opportunity to encourage further reflection and alternate interpretations of the data (Phase 6).

### Ethics

This qualitative study was approved by the Faculty of Health, Deakin University low risk ethics Committee (HEAG 114_2019) and informed written consent was obtained from all participants.

## Results

A total of 12 representatives from 11 stakeholder organisations provided consent and participated in the study. Of the four organisations that were approached and did not participate two declined due to organisational changes/re-structuring, one due to lack of capacity and one agreed but could not find a suitable time to meet for the interview. Interviews ranged from 15 to 60 min in length, with three face to face individual interviews (mean duration 46 min), one group face to face interview involving 2 participants (duration 47 min) and seventelephone interviews (mean duration 29 min).

The final sample consisted of (*n* = 12) representatives from 11 stakeholder organisations focussed on health, physical activity and/or postnatal mental health. The sample of stakeholder organisations included hospital/ clinical/ community services (*n* = 5), government agency (*n* = 3), professional associations (*n* = 2) and a non-government organisation (*n* = 1). Stakeholder organisations had local (*n* = 1), state-wide (*n* = 7) and nation-wide (*n* = 3) reach. Participant roles included policy advisors (*n* = 5), practitioner/service managers (*n* = 7) representing mental health, physical activity, physiotherapy, general practice and maternal and child health services.

**Fig. 1:**
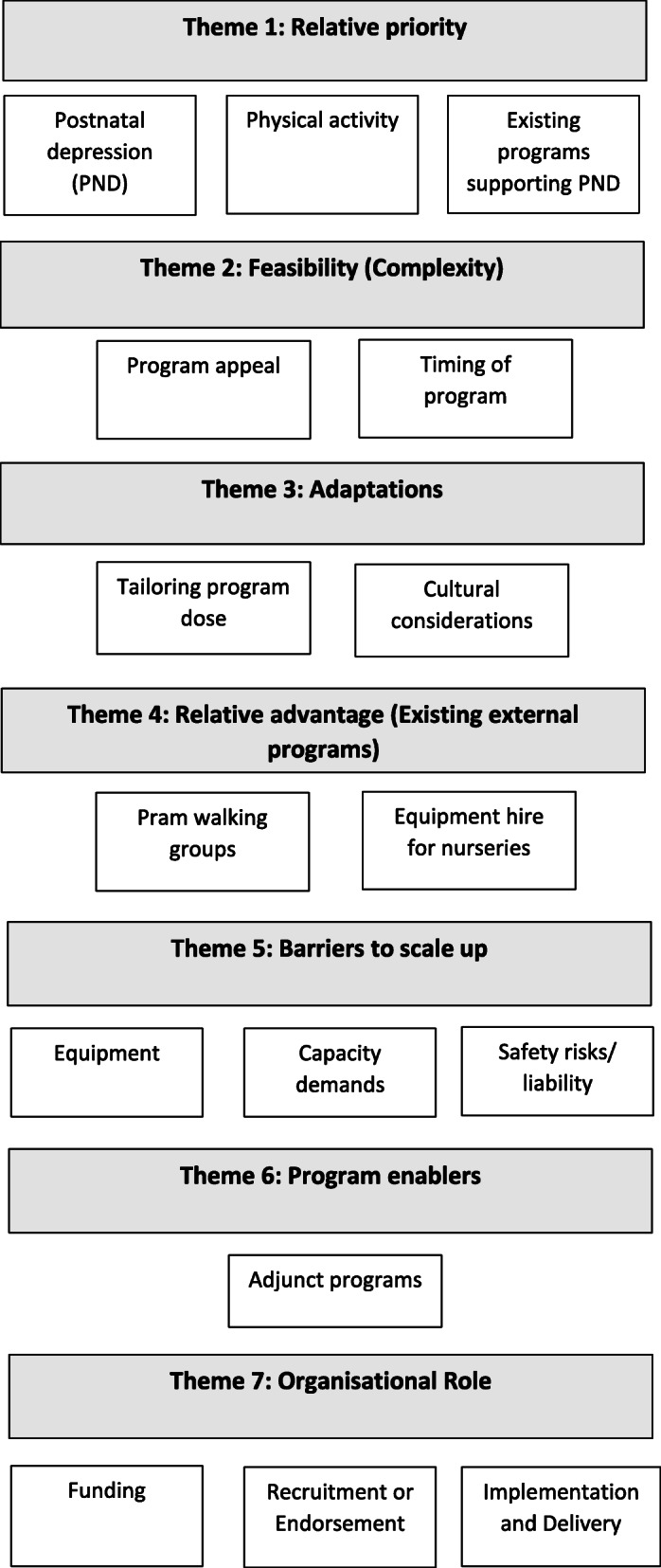
Themes and sub-themes constructed from qualitative data

To determine key stakeholder perspectives on the design and delivery of the *Mums on the Move* program, seven key themes (and 16 sub-themes) were constructed from qualitative data, guided by the Consolidated Framework for Implementation Research (CFIR) [18] and are described below (see also Fig. 1).

### Relative priority

In this theme, participants discussed postnatal depression, mental health, physical activity and current services targeting these, and whether these aligned with priorities of their organisation.

#### Postnatal depression

All stakeholder representatives stated that postnatal depression was a key priority area for their organisation. Further, a few stakeholders cited that the ongoing Australian Government reviews into mental health highlighted the need to prioritise postnatal depression.

*“There’s also obviously more broadly the royal commission into mental health services currently going on. So that indicates a priority of Government to improve the mental health system more broadly and more specifically in relation to postnatal depression”* (Stakeholder 4, Policy advisor).

#### Physical activity

Most stakeholders indicated that physical activity promotion was a priority within their organisation, particularly those working in postnatal physiotherapy who articulated the physical and mental health benefits associated with postpartum physical activity.

*“For the [physiotherapy] team, it is something that we recognise as being really important for women through pregnancy and then also postnatally. I would say we talk about it in every interaction we have with someone who’s pregnant or postnatal, whether that’s the inpatients, outpatients or group education sessions”* (Stakeholder 10, Practitioner/ service manager).

However, one stakeholder stated that physical activity was not prioritised as their organisation simply did not have the resources to promote physical activity, due to competing priorities.

*“… it’s not a priority to the extent that we would look to providing physical activity programs, but that said, part of that is because our brief is a national brief and it’s impossible to provide that kind of program on a national basis without obviously extensive resources and so we wouldn’t prioritise that”* (Stakeholder 11, Practitioner/ service manager).

#### Existing programs within the organisation supporting PND

A handful of stakeholders reported that their organisation currently had existing programs supporting women at risk of postnatal depression. These programs/initiatives included scheduled screening appointments for PND, group-based social support programs and services from maternal child health nurses such as home-based visits to women at risk.

*“… it’s a strong focus. We have a number of programs which I guess you would categorise as enhanced services, so additional visits by the nurse, home-based visits by the nurse and there may be referrals to other services, both mental health services and early parenting services”* (Stakeholder 5, Policy advisor).

Most stakeholders reported their organisation had no existing physical activity programs supporting women at risk of postnatal depression. However, one stakeholder reported that a yoga program and music therapy/ dancing program was offered to mothers experiencing PND and anxiety at their organisation. These programs were designed predominately to increase mother-baby bonding.

*“[The program] is a mother and baby program, we do singing and movement with mums. They all love that, and these are women with postnatal depression and anxiety. We’re using that mainly as a way of engaging with the baby and encouraging play and interaction, but the mums… from a physical activity point of view…they’re actually getting up and doing something so it’s quite nice”* (Stakeholder 7, Practitioner/ service manager).

### Feasibility (complexity)

Most stakeholders perceived the program as feasible for real-world implementation, although a few suggested that this may depend on factors such as cost of equipment (see ‘Barriers to scale-up’ below) and whether the program is an adjunct to existing programs (see ‘Enablers of Program’ below).

#### Program appeal

Most stakeholders suggested that the program was appealing as it breaks down the barriers postpartum women experience in relation to physical activity.

*…I think it is absolutely brilliant because I know how hard it is as a new mum to exercise…There’s always something that stops you be it sleep time or nap time or it’s too hot or the baby might need to feed- so I think if you can do anything at home, it’s going to be significantly more accessible than outside of the home environment”* (Stakeholder 8, Practitioner/ service manager).

#### Timing of program

A theme that emerged was the timing of the intervention which included both the time-point in the postpartum period that women would receive the program and the time of year the program was offered. Two stakeholders clarified that screening for PND occurred at 4 weeks postpartum, which could be an optimal time to refer to the program. One stakeholder stated that commencing the program at six to 8 weeks postpartum may be more effective than at 12 weeks postpartum as it would allow women to initiate physical activity and build their self-efficacy earlier post-childbirth.

*“.. six to eight weeks would be a good starting point because it’s when people are at that point where they’re not as overwhelmed by the baby and they’re ready to invest a little bit in their own health”* (Stakeholder 10, Practitioner/ service manager).

In addition, one stakeholder believed the feasibility of scaling up the program would also be dependent on the time of year the program was offered to participants.

*“I think particularly in the winter months, the clients would love it. I’m not sure whether in the summer months it would be as popular”* (Stakeholder 6, Policy advisor).

### Adaptations

Both sub-themes included an “equity” focus, providing suggestions on how to reduce socioeconomic and/or cultural inequalities within the program design/delivery.

#### Tailoring program dose

Two stakeholders suggested women could be provided with greater flexibility in the option of choosing the program components (e.g. equipment, web-app, text messages or online forum) they preferred, rather than providing all components to all women.

*“…there’s probably multiple solutions for people. …where some patients get the option of just getting the text messages and the park meet up but then you actually want the equipment at home so that’s a good program for you.* (Stakeholder 10, Practitioner/ service manager).

This ‘multi-pronged approach’ was further highlighted by another stakeholder as a strategy to enhance equity across different socioeconomic groups, suggesting that the most socioeconomically disadvantaged women (e.g. low income) could receive the ‘full program package’ (i.e. equipment, web-app, text messages and online forum), whilst those who are of a higher socioeconomic position (e.g. high-income) may receive just certain elements (e.g. just the web-app and online forum).

#### Cultural considerations

The importance of cultural considerations in both the intervention design and content particularly in the web-app was highlighted by one stakeholder. Cultural confinement post-childbirth was one factor that could impact on participant recruitment and timing of the intervention.

*“We have a large Chinese and Indian Community here and quite a few of them adhere to cultural confinement too so they don’t leave the house for four to six weeks... so we visit them at home until they’re ready to come to a centre. But probably that would be too early to introduce the program to them”* (Stakeholder 6, Policy advisor).

In addition, it was suggested that having the web-app content translated into other languages would make this program more culturally appropriate and reach women with or at risk of postnatal depression who are more likely to be socially isolated.

### Relative advantage (existing external programs)

#### Pram walking groups

The majority of stakeholders reported they were not aware of any similar existing programs run by other organisations. A few stakeholders, however, mentioned hearing of pram-walking groups (without being able to provide specific details of these), although it was acknowledged that such programs required a different level of involvement from participants (e.g. more structured, outdoors).

*“There was that walking one- this was a couple of years ago now, but it was pram-walking and that showed definitely, yeah, improvements in physical activity”* (Stakeholder 9, Practitioner/ service manager).

#### Equipment hire for nurseries

Although not related to physical activity or postnatal depression specifically, one stakeholder described an existing program whereby nursery equipment is offered/provided to socio-economically disadvantaged families with babies, free of hire charge. That program strategy evidently aligns with that of *Mums on the Move*, suggesting that there is precedence of free hire equipment being made available to families of low socioeconomic position.

*“There are some related programs that focus, for example, on things like nursery equipment and so forth where we fund the provision of equipment but that’s not exercise equipment specifically”* (Stakeholder 5, Policy advisor).

### Barriers to scale up

#### Equipment

A handful of stakeholders expressed concerns about the reliance on equipment within the program and the logistics/costs associated with providing the exercise equipment, being viewed as potential barriers to the scale up of this program.

*“I think the only issue would be the logistics of getting the exercise equipment in the home but if that’s not a big issue, I don’t think it would be a problem at all. I’m tipping hiring the equipment is probably fairly pricey too so that could be an issue”* (Stakeholder 8, Practitioner/ service manager).

#### Capacity demands

A few stakeholders mentioned they simply did not have the capacity to incorporate another program within their organisation as they lacked resources and were overloaded with additional programs.

*“…we’ve got to put into context is all the budget commitments for the state in regard to maternal and child health. Everything from baby bundles, to first aid courses, to checking car seats, to information sharing, to family violence and the autism screening which has just been rolled out. I can tell you that most nurses will say this looks amazing, but I’ve got no capacity [to deliver the program]”* (Stakeholder 3, Policy advisor).

#### Safety risks/liability

Some stakeholders discussed the injury risks (for mother or baby) associated with the program and the potential liability of their organisation if they were involved in the scale up of *Mums on the Move.* It was suggested that a signed document from participants would be essential to ensure organisations involved were not liable. Alternatively, one stakeholder suggested providing play pens with the exercise equipment, which would ensure young children are not at risk whilst the equipment is in use, could be one strategy to overcome this concern.

### Enablers of program

#### Adjunct programs

A few stakeholders believed that linking the *Mums on the Move* program to existing programs (i.e. ‘piggy-backing’) could facilitate program scale up. This could involve integrating the program within existing services, programs and referral systems.

### Organisational role

#### Funding

A couple of the stakeholders (including those representing non-government organisations) stated their organisation could have a role in funding the program. However, most stakeholders felt their organisation would not be able to fund it due to budget restrictions and rather would be involved in scale-up in other ways (e.g. program recruitment, endorsement). Those stakeholders further suggested that other funding bodies could include the Government (state and local), advocacy groups, community funding, sports owning bodies, grant funding and non-government organisations such as the Heart Foundation, Beyond Blue, YMCA.

#### Recruitment or endorsement (linkages and referrals)

Organisations already listed above as potential funders (e.g. state government, Beyond Blue) were also suggested by stakeholders as being organisations that potentially could be involved in endorsing the program. In addition, some stakeholders stated that referrals to the program could come from primary health care professionals.

*“So….it could be a number of different referral points or it could be GP’s in partnership with the state health departments”* (Stakeholder 9, Practitioner/ service manager).

#### Implementation/delivery

Stakeholders were asked about organisations that could potentially deliver the program, however none were suggested.

## Discussion

Key stakeholder analysis is a critical step to facilitating implementation of physical activity programs, ensuring programs are ready for real-world roll out. However, little research to date has explored key stakeholder insights to inform the design and delivery of physical activity programs, particularly home-based programs, for preventing and/or managing PND symptoms.

Overall, most stakeholders suggested that the relative priority of their organisation in targeting physical activity and/or postnatal depression was high in line with a national focus on these issues. However, no physical activity programs specifically targeting women at risk of PND were identified by stakeholders, highlighting a major gap. Although the evidence linking physical activity to PND prevention and/or treatment is understood by such organisations, little action has been taken to develop and/or implement physical activity programs for women at risk of PND. This finding is in line with calls to action by researchers and professional organisations for the implementation and translation of feasible physical activity programs for mental health into routine practice [7].

The *Mums on the Move* program was perceived by stakeholders as appealing, particularly due to its ability to overcome major barriers to postnatal physical activity. This is consistent with the views of participants (i.e. women experiencing heightened PND symptoms) in the proof of concept study [6], who suggested the flexibility and convenience of the program allowed them to be active at home, at a time that works for them, and despite unpredictable baby routines, which was highly valued. However, a few adjustments and/or considerations were suggested to further enhance feasibility for real-world scale-up. These included considering the timing of the program in regard to when PND screening is conducted (facilitating appropriate referral to the program), time of year (season/weather) and the optimal time to safely initiate physical activity after childbirth. As a consensus, providing access to the program 4–8 weeks postpartum appears to be ideal as it: 1) aligns with when mothers are routinely screened by midwives for PND (i.e. fitting with existing service delivery infrastructure, identified as critical for program scale-up [20]), and 2) aligns with recommendations for postpartum women to safely resume more intense physical activity (e.g. [21]); and 3) is an appropriate time for mothers to focus more on their own health and initiate physical activity routines.

Additionally, suggested adaptations/alterations took on an equity lense as they aimed to reduce socioeconomic and cultural inequalities within the program. This was via either offering a different program dose based on either 1) socioeconomic position (e.g., all women may receive the web-app and text messaging components but only low-income women may be offered treadmill hire free of charge); or 2) ensuring the program content (particularly the web-app) was translated into various languages. The importance of women being able to choose which components of the program they wanted (rather than a one-size-fits-all) was also highlighted. Given that PND is socioeconomically skewed [22] and that those from culturally and linguistically diverse groups are also at heightened risk [23], it is important that when designing interventions targeting improvements in PND, programs consider these high-risk groups.

Despite no existing similar home-based physical activity programs being identified for the prevention or treatment of PND by the interviewees, it was suggested that *Mums on the Move* aligned with other existing programs or initiatives in the postnatal space. This included a Government funded program, which provides large equipment free of hire (i.e. nursery equipment such as cots) to disadvantaged families. Given that the cost and logistics of exercise equipment hire was identified by stakeholders as a potential barrier to scale-up, this demonstrates that provision of free equipment hire for postnatal women already occurs within existing Government systems. Pram walking groups were also suggested by stakeholders to align with the *Mums on the Move* program. Although limited evidence has shown pram walking groups may improve PND symptoms in a research setting [24], pram walking groups are unable to overcome the barriers to physical activity that *Mums on the Move* is able to (e.g. flexibility, working with baby routines). Yet, these groups could be used as an adjunct strategy within the *Mums on the Move* program (e.g. providing details of existing pram walking groups via the web-app for when women have established an exercise routine/enhanced self-efficacy to be active). This could be particularly useful as a maintenance strategy for when the exercise equipment is removed after the initial 12-week ‘equipment’ period.

Another barrier to scale-up identified by stakeholders was organisational capacity/competing priorities. This finding is consistent with those of a systematic review investigating barriers to scaling up health programs [25]. Most organisations involved in the current study were responsible for running multiple programs targeting various population groups, behaviours and health needs. Thus, this underscores the importance of ensuring 1) physical activity and postnatal depression are perceived as high-priority by relevant organisations; 2) proposed programs are supported by evidence and effectively ‘packaged’; 3) programs are co-designed with a range organisations potentially responsible for implementation [25]. Further, as already mentioned, ensuring programs are developed with potential to be embedded within existing infrastructure or services [20, 25] may also overcome the barrier of organisational capacity and competing priorities, and rather facilitate implementation. This strategy was also highlighted by stakeholders in the current study whereby it was suggested that ‘piggy-backing’ on existing maternal health programs may facilitate the implementation of *Mums on the Move.* Finally, few organisations suggested they would be involved in the funding or delivery of the program and most would be referring organisations. Thus, more evidence of cost effectiveness of such programs is required before organisations might invest in this type of program.

This study has a number of strengths and limitations. Strengths include the recruitment of representatives from a broad range of stakeholder organisations relevant to physical activity and/or postnatal health/mental health, who were equipped to provide expert opinion on the potential scalability of the program. Further, the qualitative study design enabled rich and detailed insights on a previously under-studied research topic. The interview schedule and coding framework were guided by existing implementation and scale up frameworks for informing program implementation and scale-up of physical activity programs (i.e. CFIR, PRACTIS) [10, 18] ensuring that key aspects of program implementation were explored while allowing for new issues to be raised through the use of open ended question and inductive coding. The interviewer had both intimate knowledge of the program (i.e. the designer/developer) and experience as being a mother of young children, enabling a greater level of detail when probing and/or responding to questions during interviews. Detailed notes were taken immediately following each interview regarding key issues arising which were used alongside the interview transcripts in the analysis. While the final themes identified were agreed by the authors involved in data collection and analysis, these were not taken back to participants for member checking in the interest of time and reducing participant burden and this is acknowledged as a limitation. Other limitations include the small sample size (*n* = 12) which may have limited the range of stakeholder perspectives and insights provided. For example, a couple of major mental health support organisations declined to participate due to not having capacity at the time. Further, the perspectives of maternal child health services located specifically in socio-economically disadvantaged neighbourhoods, as well as organisations with the potential to fund such a program may be underrepresented. The focus on one specific physical activity program may limit the transferability of the findings to physical activity programs offered to women in other settings and contexts. However, many of the issues identified such as the fit with existing programs, program complexity, appeal, timing, adaptations, cultural considerations, funding and referrals are likely to be important in the scale up of many physical activity programs. We acknowledge there is the potential for social desirability bias given the interviewer was also the designer / developer of the intervention, although steps were taken to minimise this (described earlier). Stakeholders (participants) were not known to MT, except for one. We acknowledge that some interviews were short in duration, and this may reflect the fact that some participants were in busy policy positions and had more limited time to contribute. A one-page brief of the project was however provided prior to the interview to provide necessary background information.

## Conclusion

To date, limited implementation research in the area of physical activity and PND has been conducted. This study demonstrates the importance of working with stakeholder organisations in the co-design of physical activity programs to promote mental health in postnatal populations. Findings not only inform the scale-up and implementation of the *Mums on the Move* program, but also inform the broader researcher community on key program considerations, barriers and facilitators for implementing public health programs. Despite being appealing such programs should consider ensuring: 1) Programs address inequalities in health (socio-economic and cultural); 2) Programs are designed with potential to embed within existing infrastructure, services and systems; 3) Organisations responsible for funding, endorsement and delivery are identified early on. Given that most stakeholders suggested that the potential role their organisation would play in scale-up and implementation of *Mums on the Move* would be in referral/endorsement of the program, further exploration of potential organisations as funding and/or delivery agents is needed.

## Supplementary Information

**Additional file 1: Table S1.** Interview schedule and related CFIR and PRACTIS constructs

## Data Availability

The datasets analysed during the current study are not publicly available due to ethical restrictions (participants have not consented to the use of their data for purposes other than those for which they originally consented). Should a researcher request the data for a particular purpose, an ethically compliant (anonymised) dataset may be made available via the lead author upon approval by the Deakin University Human Research Ethics Committee. Requests can be emailed to: research-ethics@deakin.edu.au

## Abbreviations

PA: Physical Activity
PND: Postnatal Depression
CFIR: Consolidated Framework for Implementation Research
PRACTIS: PRACTical planning for Implementation and Scale-up
SRQR: Standards for Reporting Qualitative Research
NGO: Non-Government Organisation
