# Supplementary material for: Key stakeholder perspectives on the development and real-world implementation of a home-based physical activity program for mothers at risk of postnatal depression: a qualitative study
Source: BMC Public Health. 2021 Feb 16;21:361. doi: 10.1186/s12889-021-10394-8 (PMC7885569; doi:10.1186/s12889-021-10394-8)
Supplement: Supplementary file 1 — Additional file 1: Table S1. Interview schedule and related CFIR and PRACTIS constructs [file 12889_2021_10394_MOESM1_ESM.docx]

**Supplementary table 1.** Interview schedule and related CFIR and PRACTIS constructs

| **Interview question** | **CFIR construct** | **PRACTIS guide construct** |
| --- | --- | --- |
| 1. Could you tell me a little bit about your role here at X organisation? | N/A | N/A |
| 2. Would you say PND is a priority for your organisation?   - If not, why not? (e.g. competing priorities, limited capacity, vision of the organisation) - If yes, do you currently have programs/initiatives supporting women at risk of PND? (if yes – what are these?) | Inner setting   - Implementation climate: Relative priority |  |
| 3. Would you say Physical Activity is a priority for your organisation?   - If not, why not? (e.g. competing priorities, limited capacity, vision of the organisation) - If yes, do you currently have PA programs/initiatives supporting women at risk of PND? (if yes – what are these?) | Inner setting   - Implementation climate: Relative priority |  |
| 4. In what way, if any, does MOTM program *fit* with the work of your organisation (*prompts: align with any existing policies, initiatives, programs*) *e.g. What about the [INSERT EXAMPLE POLICY/PROGRAM FROM THE ORGANISATION] policy/program?* | Inner setting   - Implementation climate: Compatibility |  |
| 5. How *feasible* do you think MOTM would be to implement at scale? (How *complicated* is the program?) | Intervention characteristics   - Complexity | **Step 1:** Characterise parameters of implementation setting   - Intervention factors (all levels)   **Step 4:** Address potential barriers to implementation   - Formative evaluation (stakeholder engagement, participatory research) – Provider level |
| 6. Are there any *changes or alterations* you would make to the MOTM program to make it fit or work effectively in your setting? If yes, what are these? | Intervention characteristics   - Adaptability | **Step 1:** Characterise parameters of implementation setting   - Intervention factors (all levels)   **Step 4:** Address potential barriers to implementation   - Formative evaluation (stakeholder engagement, participatory research) – Provider level |
| 7. *Relative advantage* – Do you know of any other similar existing programs that target physical activity and PND? (*e.g. home-based programs? PA programs? PND programs?*) (ask for names of these).  Program name:  Who runs it:  How is it delivered/structured:  How would you rate that program: | Intervention characteristics   - Relative advantage | **Step 1:** Characterise parameters of implementation setting   - Intervention factors (all levels) |
| 8. *Evidence* – What information do you need to convince you or your organisation of the benefits of being involved in MOTM? | Intervention characteristics   - Evidence strength and quality |  |
| 9. What do you see as *barriers* to the scale up of this program – both relevant to your organisation and in general? (*Prompts: e.g. funding, external policies/incentives, policy, politics, community readiness for prevention, appropriate way to target PND?*) |  | **Step 3:** Identify implementation barriers and facilitators   - Provider level (implementer beliefs) |
| 10. What would help enable the scale up of this program? (*Prompts: e.g. goals/vision, shared decision making and vision, organisational support for implementation, networks/communication, existing supports [online resources, marketing], organisation commitment*) |  | **Step 3:** Identify implementation barriers and facilitators   - Provider level (implementer beliefs) |
| 11. Would your organisation consider being a partner on the MOTM program? If yes, what role, if any, could you see [INSERT ORGANISATION NAME HERE] playing in the implementation of the MOTM program if it was scaled up in Victoria? E.g.   - **funding**, *> lead to question on funding (how?)* - **endorsing (e.g. referral, or advertising/informing),** *> lead to question on referral/dissemination (how? - clarify here if they could disseminate online given the success we had previously via online/social media recruitment of this population group). How would you envisage mums with PND could be recruited (either referral or informing) into the program if this was scaled up?* - **implementing/delivering***.> lead to question on delivery of the intervention (how?)* |  | **Step 1:** Characterise parameters of implementation setting   - Intervention population - Individual level - Implementers - Provider level   **Step 2:** Identify and engage key stakeholders across multiple levels within the delivery system   - Intervention funding/responsibility - Intervention dissemination/spread - Intervention host/deliver – implementation) |
| 12. Do you have any ideas on how we could *work together* in future (whether it be on the MOTM program or other)? | N/A | N/A |
| 13. Can you see a *role* for any *other organisations* and if so who/how? |  | **Step 2:** Identify and engage key stakeholders across multiple levels within the delivery system   - Intervention funding/responsibility - Intervention dissemination/spread - Intervention host/deliver – implementation) |
| 14. Is there anything else you would like to add? | N/A | N/A |
| 15. Would you be happy for me to keep you up to date with the research and with the outcomes from the funding we apply for? | N/A | N/A |
